# Assessing Acerola Powder as Substitute for Ascorbic Acid as a Bread Improver

**DOI:** 10.3390/foods11091366

**Published:** 2022-05-08

**Authors:** Maria Franco, Mayara Belorio, Manuel Gómez

**Affiliations:** Food Technology Area, College of Agricultural Engineering, University of Valladolid, 34004 Palencia, Spain; mariafrancomarcos@gmail.com (M.F.); beloriom@gmail.com (M.B.)

**Keywords:** bread, acerola, ascorbic acid, oxidant, wholemeal

## Abstract

Bread is one of the most widely consumed products in the world. The use of oxidants is common in bread production, but consumers are demanding products with less additives. Acerola is the fruit with the highest ascorbic acid content and, once dried, it can be used as an oxidant in baking. The use of acerola powder in bread making and its effect on bread quality is studied in this article and compared with the addition of ascorbic acid. For this purpose, flour properties and dough behaviour were analysed with a farinograph and an alveograph. Breads were elaborated with white wheat flour and wholemeal flour; specific volume, loaf height, weight loss, texture, colour, and cell structure were analysed. Acerola powder had similar effects to ascorbic acid: it increased the alveographic strength and the tenacity of the doughs without reducing extensibility; it incremented dough development time (DDT) and dough softening; it increased the specific volume of white wheat breads, and it reduced the hardness of white and wholemeal breads, without significant changes in crust or crumb colour. Therefore, acerola powder can be a natural alternative to the use of ascorbic acid as an improver in bread making.

## 1. Introduction

Bread has been considered a staple product that contributes substantially to the daily intake of basic nutrients for human nutrition [1]. The gluten present in wheat flour plays an essential role in the rheology of bread doughs and in their ability to retain the gases formed during fermentation [2,3]. The use of oxidant agents is frequent in the baking industry to strengthen the gluten network and improve the quality of breads [4]. This is due to the formation of disulphide bonds from the hydrogen sulphide groups present in the protein chains [5]. One of the most widely used oxidants for this purpose has been potassium bromate. However, its use in bakery products is currently forbidden in many countries due to its carcinogenic effects [6]. 

Nowadays, ascorbic acid (AA) is widely used as an oxidant to strengthen the gluten network. Its chemical-based version is the most used in food formulation; however, AA is a natural substance that can be found in fruits and vegetables [7]. During dough mixing, and in the presence of oxygen, AA is modified to its oxidant form, dehydro-L-ascorbic acid, due the presence of a natural enzyme presented in wheat flour (ascorbic acid oxidase). As a result, new disulphide bonds are generated; these contribute to maintaining the stability between gluten proteins and to increasing the gluten strength. This stability produces breads with a higher loaf volume and a finer and more uniform crumb structure [4,8].

In wholemeal breads the presence of bran and germ in the flour reduces gluten strength, gas retention and bread volume. In these cases, the use of oxidant agents, such as AA, has proven to be effective [9,10,11,12].

Nowadays, some people are trying to reduce the consumption of food with additives. In response to this demand for a clean label, i.e., food made from natural ingredients without the addition of artificial ingredients or preservatives, producers are facing the need to replace them with natural substitutes [13].

Acerola (*Malpighia emarginata* DC.) is a fruit native from the West Indies and tropical South America and it has the highest vitamin C content of all fruits [14]. Acerola also contains bioactive substances, such as carotenoids and phenolic compounds, with high antioxidant capacity [15,16]. Acerola powder (AP) has already been suggested to improve the quality of gluten-free breads [17]. In this case, the improvement was not due to the redox reactions and their effects on proteins. However, the use of AP in wheat breads and its potential effects on dough rheology and bread quality have never been studied. 

The aim of this study was to test the ability of acerola powder to substitute ascorbic acid in baking. For this purpose, white wheat (WF) and a wholemeal (WWF) control flours with different concentrations of AA and AP were evaluated through farinograph and alveograph (only WF) analyses. Then, WF and WWF breads were elaborated and the effect of different concentrations of AP (0.05% and 0.1%) was evaluated, in comparison to AA (0.01%) on the specific volume, loaf height, texture, colour and cell structure of control breads.

## 2. Materials and Methods

### 2.1. Materials

Pan breads were elaborated with two different flours, WF (ash 0.8%, falling number 340) and WWF (ash 1.5%, falling number 340), milled in stone milling and supplied by Molinos del Duero—Carbajo Hermanos S.A. (Zamora, Spain). Alveograph and farinograph flour properties are shown in Table 1. AA (Panreac Quimica S.A.U., Barcelona, Spain) and AP (15.62 g/100 g ascorbic acid) (Eltabia GmbH, Münster, Germany) were used as oxidant agents. Other ingredients were instant yeast (Dosu Maya Mayacilik A.S., Istambul, Turkey), salt (Disal, Madrid, Spain), and potable water.

### 2.2. Methods 

#### 2.2.1. Flour Analysis and Dough Quality

Alveograph measurements of WF were performed using an Alveograph MA 82 (Chopin, Tripette et Renaud, Villeneuve La Garenne, France) and the standard AACC method 54-30 [18]. The parameters obtained were: dough tenacity (P), dough extensibility (L), dough strength (W), and the ratio between P and L (P/L). 

The farinograph test of both flours WF and WWF flours were performed with a Farinograph-AT (Brabender, Duisburg, Germany) according to the AACC method 54-21 [19]. The parameters obtained were: water absorption, dough development time (DDT), stability, and dough softening. 

Alveograph and farinograph were performed in duplicate.

#### 2.2.2. Bread Making

Pan breads were elaborated using a straight dough method. The following ingredients (g/100 g flour basis) were used: water (estimated according to the farinograph results of water absorption (Table 1)), instant dry yeast (2 g/100 g) and salt (1.8 g/100 g). Water temperature was calculated to achieve a final dough temperature of 23 °C. Two control breads were elaborated, one with WF (Control) and another with WWF (WControl). AA (0.01 g/100 g) and AP (0.05 g/100 g and 0.1 g/100 g) were added to each control formulation to produce other six bread formulations: AA 0.01%, AP 0.05%, AP, 0.1%, WAA 0.01%, WAP 0.05%, and WAP 0.1%. 

All dry ingredients, including the instant dry yeast and salt, were placed together and, then, water was added. The ingredients were mixed using a spiral kneader (Ferneto, Vagos, Portugal). Formulations containing WF were mixed for 20 min and those with WWF for 25 min, until the complete development of the gluten network. The dough was divided in pieces of 300 g, which were manually rounded and set aside for a 10 min rest. Later, the samples were moulded into bars (230 mm long) using a mechanical former (Subal, Paterna, Spain). For each elaboration, samples were placed in an aluminium pan (232 mm × 60 mm × 108 mm). Fermentation was conducted for 120 min at 30 °C and 75% of relative humidity. Breads were baked at 220 °C for 25 min. 

After baking, breads were removed from the moulds and placed to rest and to cool at room temperature for 1 h. Later, they were packed in plastic bags, properly sealed, and stored for 24 h until further analysis. All bread formulations were performed in duplicate.

#### 2.2.3. Bread Characteristics

Breads height was evaluated using a digital calliper. Measurements were taken at the centre of the sample. 

Weight loss was calculated as the difference between the weight of the dough before baking and the weight of breads after 24 h. Results were expressed as percentage. 

Bread final volume was obtained with a Volscan Profiler volume analyser (Stable Microsystems, Surrey, UK). Breads specific volume was calculated through the relation between bread volume and bread weight (cm^3^/g). 

Bread height, weight loss, and specific volume were measured in four breads of each elaboration (4 × 2).

Crumb texture was measured through a Texture Profile Analysis (TPA) with a double compression test and a TA-XT2 texture analyser (Stable Microsystems, Surrey, UK). Bread loaves were sliced at the centre with a thickness of 30 mm. One central slice from four breads of each elaboration were evaluated (1 × 4 × 2). For the analysis, a 25 mm diameter cylindrical probe penetrated 50% of the depth of each slice using a trigger force of 5 g and a test speed of 1 mm/s crumb. The application conditions of the TPA method were as follows: pre-test speed 2 mm s^−1^, test speed 1 mm s^−1^, post-test speed 2 mm s^−1^. Hardness was obtained and cohesiveness and resilience were calculated. Crumb hardness was also evaluated seven days after baking to obtain the increase in hardness (Δ), which was calculated as the difference between the results of hardness at day seven and day one, expressed as a percentage.

Crust and crumb colours were measured with a colorimeter (PCE Instruments, Hochsauerland, Germany), a D65 illuminant with a 2° Standard Observer. Crust colour was measured in two different points in the surface of four breads of each elaboration (2 × 4 × 2). Crumb colour was determined in the centre of the middle slice of each bread loaf (4 × 2). Results were expressed in the CIE L* a* b* colour space, and the parameters obtained were L* (whiteness/darkness), a* (redness/greenness), and b* (yellowness/blueness).

#### 2.2.4. ImageJ

Central bread slices of 30 mm thick were scanned using an HP scanner (HP ScanJet 300, Palo Alto, Santa Clara, CA, USA). Fractions of 30 mm × 30 mm area were taken from the centre of each image and were analysed in ImageJ software version 1.53e (National Institutes of Health, Bethesda, MD, USA) using an 8-bit image type. The parameters obtained were total pores, mean area, and cell size. One slice from two breads of each elaboration were analysed (2 × 2). 

#### 2.2.5. Statistical Analysis

Analysis of variance (ANOVA) was used to study the differences between dough and bread characteristics. Tukey’s HSD was used to describe means with 95% confidence. Analysis was performed using Statgraphics Centurion 18 V18.1.13 software (StatPoint Technologies, Warrenton, VA, USA).

## 3. Results and Discussion

This section may be divided by subheadings. It should provide a concise and precise description of the experimental results, their interpretation, as well as the experimental conclusions that can be drawn.

### 3.1. Rheological Properties

The results of the farinograph and alveograph analyses are presented in Table 1. No differences were observed between the absorption of WF with AA or AP. However, the absorption of those flours was lower than the control. The absorption of WWF was higher than WF absorption and only the highest concentration of AP increased this value. All the absorption differences were very small and did not reach 1% in any case. These data are in agreement with the observations made by Gelinas and McKinnon [20] and may be related to the early changes that ascorbic acid undergoes during the beginning of mixing [21]. Tozzati et al. [4] also found minimal changes in the rheology of doughs with AA, but depending on the wheat variety these changes were not significant in many cases. 

DDT increased for both WF and WWF with the addition of AA and AP. However, in general, no significant differences were found between WF and WWF doughs with the addition of AA or AP. Only in the case of WF, AP 0.1 had a lower DDT than AA 0.01. Stability was clearly increased with AA and AP in the case of WF, as also observed by Gelinas and McKinnon [20] with the addition of AA. Additionally, AP and AA increase dough softening. Considering stability, no significant differences were observed between AA and AP, but dough softening was higher for AP, at either concentration, compared to AA. In the WWF, AA and AP reduced stability and increased dough softening. In general, both DDT and stability are related to the quality of the protein and gluten network [22]. Therefore, the changes in the results of the farinographic analysis were generated by the presence of AA, due to the formation of disulphide bonds, which help to strengthen the gluten network [23]. 

Regarding the alveographic analysis, AA and AP increased W and P, and reduced L, increasing P/L.However, L reduction only was significant with AA. Maforimbo, Nguyen, and Skurray [24] also observed an increase in extension resistance with AA, but no significant differences were found in extensibility, while analysing doughs with an extensograph. These results were motivated by the oxidation of L-ascorbic acid to dehydro-ascorbic acid, which became the oxidising agent. This reaction promotes the formation of disulphide bonds that strengthen the gluten network [25,26]. No significant differences were found between AA and AP, at either concentration, in L or P/L values. Therefore, the addition of AP could have a similar effect to AA, due to its high AA content [14]. No significant differences were found between small concentrations of AP and AA in P or W values, but high concentration of AP developed greater values than the lower concentration. This indicates that it would be necessary to adjust the amount of AP according to the target, in the same way as it would be carried out for the AA [27].

### 3.2. Bread Properties

**Figure 1:**
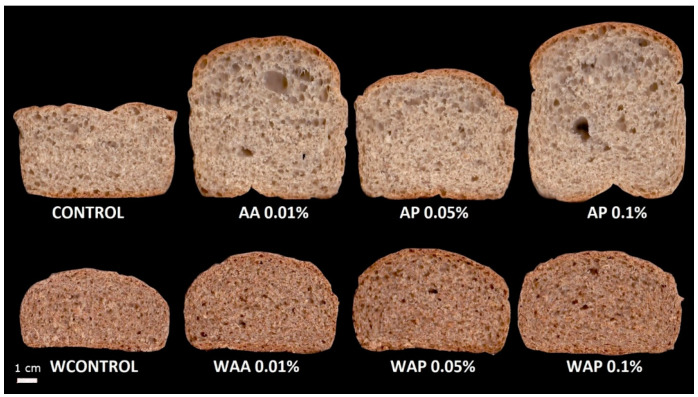
Bread slices scanner images elaborated with: Control: wheat flour; AA 0.01%: wheat flour with 0.01% ascorbic acid; WF AP 0.05%: wheat flour with 0.05% acerola powder; AP 0.1%: wheat flour with 0.1% acerola powder; WControl: whole wheat flour; WAA 0.01%: whole wheat flour with 0.01% ascorbic acid; WAP 0.05%: whole wheat flour with 0.05% acerola powder; WAP 0.1%: whole wheat flour with 0.1% acerola powder.

The height and specific volume of breads are presented in Table 2. Ascorbic acid and AP increased the height of breads made with WF and WWF. Ascorbic acid and AP 0.1 also increased specific volume in WF breads. This increase in specific volume is not significant in WWF breads. No significant differences were observed between the height and the specific volume in breads with AA or with any AP concentration, but values of AP 0.1 were higher than AP 0.05. These effects can also be observed in Figure 1. The effect of AA on the volume of WF breads has already been proved in other studies [25,27], and it has been attributed to the reinforcement of the gluten network and its better capacity to retain the gas formed during fermentation at the beginning of baking. In addition, Beghin et al. [28] observed that this effect was greater when yeast was incorporated, as in the bread making process. This effect was attributed to the presence of small amounts of glutathione and its interaction with AA in redox reactions. This interaction was also observed by Every et al. [29] while studying different millstream flours. In WWF breads, results were not evident and, while some authors observed increases in specific volume with the addition of AA [10,11,12], others did not [30]. This suggests that for WWF breads, the effect of AA would depend on the type of bread making process and the flour used. The differences in glutathione and oxidized glutathione content, which in the case of bran and germ is higher than in WF, also affect it [29]. A similar effect of AP on breads was reported by Boz and Karaoglu [31] who used Rosehip powder, another natural product with a high AA content. These authors observed a significant increase in the specific volume of the breads, but they used higher levels of powder (2.5%), almost 200 times more than the levels used in this study with AP. This confirms the potential of natural products rich in AA as substitutes for pure AA—obtained through chemical processes—as a bread improver.

As regards cell structure (Table 2), it was observed that the addition of AA to WF bread did not show significant differences with respect to the total number of pores. However, it showed a lower mean area, which may be related to the smaller average pore size compared to the control. As a result, these breads had a more closed and homogeneous cell structure. The addition of AP to breads with WF did not produce significant differences compared to the control in any of the parameters evaluated. Similar results were observed by Sapirstein, Roller, and Bushuk [32] with different oxidants. The effect of AA and AP on the gluten network and its strength is responsible for the changes in cell structure. The increase in the strength of the gluten network reduces the coalescence phenomenon between the gas bubbles, usually resulting in smaller cells [33]. The larger pore area in the control breads is remarkable, despite the fact that the breads have a lower specific volume. This may indicate that the solid part of the crumb is more expanded in breads with AP and AA.

In the case of the WWF, breads with the lowest concentration of AP did not differ from the control. However, AA and AP 0.01 breads showed a lower total number of pores and a higher cell size compared to the control. Moreover, the mean area did not show significant differences between breads. Breads from WWF had a larger mean area, despite having a smaller volume. This may be due to the optical effect of the presence of bran, which has a dark colour and can be recognised as a cell in the image processing. These breads also had a larger cell size, which is in concordance with the observations of Packkia-Doss, Chevallier, Pare, and Le-Bail [34], when wheat bran was added during baking. This effect may therefore be due to the weakness of the gluten network, both because of the lower gluten content of these flours and the disruption of the gluten network caused by the bran particles [9]. For this reason, it might be necessary to increase the concentration of AP in WWF breads to obtain significant effects.

Table 3 presents the results for texture; they were clearer than those for specific volume. A reduction in hardness (near to 50%) was observed when AA and AP were incorporated in both WF and WWF breads, with no significant differences between the breads with the oxidants. In general, bread hardness is correlated with specific volume [35], so the lower hardness in breads with AA or AP could be related to the higher volume or the height of these breads. In fact, WWF breads, with a lower specific volume have much higher hardness values than WF breads [36]. Zghal et al. [33] also observed that the stronger the gluten network, the greater the volume of the breads, so the cells became narrow and the hardness decreased, thus increasing the extensibility of the crumb. The incorporation of AP in WWF breads increased the values of cohesiveness compared to the control. No significant differences were observed in resilience between WF and WWF breads. Neither was any effect observed in the increased hardness over time. Gujral et al. [36], in a study of breads made with barley flour, also found a reduction in the hardness of fresh breads with the addition of ascorbic acid, but they did not observe an effect on hardness after storage, as in our case. This seems logical because amylopectin retrogradation has a central role in bread staling [37] and AA modified the protein but not the starch structure. 

In general, no significant differences between the crust colours of the breads were observed, neither in WF nor in WWF breads (Table 4). Only in the case of the WF, breads with the lower amount of AP presented higher L* and b* values than the control. Likewise, no differences were observed in crumb colour between WF and WWF control breads when AA or AP were incorporated. In general, crust colour is related to Maillard reactions and sugar caramelisation, as the external surface of the bread is sufficiently hot for these reactions to occur [38]. Therefore, the addition of low quantities (less than 0.1%) of AA or AP, which do not modify the Maillard reactions, should not affect the colour, as has been shown. However, as the temperature does not exceed 100 °C inside the breads, the changes in colour were more related to the colour of the ingredients [39]. In this case, despite acerola red colour [40] and the pinkish colour of the flours, low quantities of this ingredient did not affect crumb colour. This is important because, if other natural products with a lower concentration of AA are used, higher concentrations are necessary to change the colour of the crumb [31,41], influencing the consumer’s perception.

## 4. Conclusions

Acerola powder may function as a bread improver, both in WF and WWF breads, due to its high AA content. It strengthens the gluten network and the gas retention capacity during fermentation due to its oxidising effect. It also increases DDT and dough softening in farinographic analysis. In addition, it can increase the specific volume of breads and reduce their hardness, without modifying the colour of the crumb or the crust. Unlike other natural ingredients with a lower concentration of AA, AP can replace AA, without affecting physical, textural, and rheological characteristics of breads and produce high quality clean labelled breads. 

## Figures and Tables

**Table 1:** Farinographic and alveographic properties of doughs.

Flour	Absorption (%)	DDT (seconds)	Stability (seconds)	Dough Softening (FU)	P (mm H_2_O)	L (mm H_2_O)	P/L	W (J × 10^4^)
Control	60.5 ± 0.0 b	355.0 ± 28.3 a	485.5 ± 0.7 d	65.0 ± 1.4 a	86.7 ± 0.5 a	81.4 ± 0.8 b	1.1 ± 0.0 a	219.0 ± 0.0 a
AA 0.01%	60.2 ± 0.1 a	547.5 ± 10.6 d	700.5 ± 5.0 f	80.5 ± 0.7 c	112.3 ± 1.4 bc	75.8 ± 0.6 a	1.5 ± 0.0 b	321.0 ± 4.2 b
AP 0.05%	60.0 ± 0.0 a	519.0 ± 0.0 cd	666.0 ± 19.8 ef	93.5 ± 2.1 d	107.3 ± 3.3 b	79.9 ± 3.7 ab	1.3 ± 0.1 b	316.5 ± 0.7 b
AP 0.1%	60.0 ± 0.0 a	513.0 ± 9.9 c	683.0 ± 8.5 ef	92.5 ± 0.7 d	114.7 ± 1.4 c	77.6 ± 0.8 ab	1.5 ± 0.0 b	333.0 ± 4.2 c
WControl	68.0 ± 0.1 c	420.5 ± 16.3 b	338.5 ± 19.1 c	69.5 ± 3.5 b	-	-	-	-
WAA 0.01%	68.6 ± 0.1 e	519.0 ± 5.7 cd	250.5 ± 6.4 a	102.5 ± 0.7 e	-	-	-	-
WAP 0.05%	68.0 ± 0.0 c	527.5 ± 9.2 cd	270.0 ± 4.2 ab	102.0 ± 0.0 e	-	-	-	-
WAP 0.1%	68.3 ± 0.1 d	532.5 ± 7.8 cd	281.0 ± 7.1 b	103.0 ± 0.0 e	-	-	-	-

Control: wheat flour; AA 0.01%: wheat flour with 0.01% ascorbic acid; AP 0.05%: wheat flour with 0.05% acerola powder; AP 0.1%: wheat flour with 0.1% acerola powder; WControl: whole wheat flour; WAA 0.01%: whole wheat flour with 0.01% ascorbic acid; WAP 0.05%: whole wheat flour with 0.05% acerola powder; WAP 0.1%: whole wheat flour with 0.1% acerola powder. Values with the same letter in the same parameter do not present significant differences (*p* < 0.05); -: not available.

**Table 2:** Bread specific volume and image analysis.

Flour	Specific Volume (cm^3^/g)	Bread Height (mm)	Total Pores	Mean Area	Cell Size
Control	2.80 ± 0.10 bc	53.50 ± 4.60 b	348.50 ± 4.24 c	372.55 ± 10.174 bc	1.07 ± 0.01 b
AA 0.01%	3.55 ± 0.58 de	75.10 ± 9.10 cd	369.00 ± 21.21 c	289.84 ± 8.00 a	0.79 ± 0.02 a
AP 0.05%	3.38 ± 0.01 cd	66.87 ± 0.82 c	343.25 ± 35.71 bc	334.74 ± 37.66 b	0.99 ± 0.21 b
AP 0.1%	4.05 ± 0.44 e	82.64 ± 5.47 d	329.50 ± 11.31 bc	331.27 ± 1.42 ab	1.01 ± 0.05 b
WControl	2.05 ± 0.04 a	41.24 ± 0.38 a	336.00 ± 26.16 bc	433.43 ± 25.07 de	1.29 ± 0.03 c
WAA 0.01%	2.40 ± 0.10 ab	55.49 ± 2.37 b	265.25 ± 2.47 a	405.68 ± 1.03 cd	1.53 ± 0.02 d
WAP 0.05%	2.50 ± 0.09 ab	53.00 ± 0.25 b	304.00 ± 2.12 ab	398.48 ± 12.31 cd	1.31 ± 0.05 c
WAP 0.1%	2.36 ± 0.00 ab	54.35 ± 0.15 b	286.25 ± 6.01 a	451.62 ± 24.95 e	1.60 ± 0.05 d

Control: wheat flour; AA 0.01%: wheat flour with 0.01% ascorbic acid; AP 0.05%: wheat flour with 0.05% acerola powder; AP 0.1%: wheat flour with 0.1% acerola powder; WControl: whole wheat flour; WAA 0.01%: whole wheat flour with 0.01% ascorbic acid; WAP 0.05%: whole wheat flour with 0.05% acerola powder; WAP 0.1%: whole wheat flour with 0.1% acerola powder. Values with the same letter in the same parameter do not present significant differences (*p* < 0.05).

**Table 3:** Textural properties of breads.

Flour	Hardness (N)	Cohesiveness	Resilience	Δ Hardness (N)
Control	9.81 ± 1.27 c	0.77 ± 0.01 ab	47.99 ± 1.53 ab	1.50 ± 0.52 a
AA 0.01%	4.99 ± 2.1 a	0.79 ± 0.02 b	49.56 ± 3.02 b	1.13 ± 0.42 a
AP 0.05%	6.03 ± 0.38 ab	0.79 ± 0.01 b	49.31 ± 0.87 b	1.86 ± 0.6 a
AP 0.1%	3.64 ± 1.53 a	0.79 ± 0.01 b	48.62 ± 2.84 b	1.17 ± 0.63 a
WControl	17.80 ± 0.49 d	0.74 ± 0.01 a	43.73 ± 0.73 a	1.25 ± 0.32 a
WAA 0.01%	9.89 ± 2.32 c	0.77 ± 0.02 ab	46.53 ± 2.65 ab	1.23 ± 0.66 a
WAP 0.05%	8.94 ± 0.97 bc	0.78 ± 0.002 b	47.35 ± 0.62 ab	1.35 ± 0.31 a
WAP 0.1%	9.45 ± 1.08 c	0.78 ± 0.005 b	46.88 ± 1.13 ab	1.38 ± 0.37 a

Control: wheat flour; AA 0.01%: wheat flour with 0.01% ascorbic acid; AP 0.05%: wheat flour with 0.05% acerola powder; AP 0.1%: wheat flour with 0.1% acerola powder; WControl: whole wheat flour; WAA 0.01%: whole wheat flour with 0.01% ascorbic acid; WAP 0.05%: whole wheat flour with 0.05% acerola powder; WAP 0.1%: whole wheat flour with 0.1% acerola powder. Values with the same letter in the same parameter do not present significant differences (*p* < 0.05).

**Table 4:** Bread crust and crumb colour.

	Crust			Crumb		
Flour	L*	a*	b*	L*	a*	b*
Control	51.89 ± 4.30 a	14.08 ± 1.07 bc	22.81 ± 1.87 a	60.47 ± 2.37 c	4.48 ± 0.04 b	14.84 ± 0.68 a
AA 0.01%	56.86 ± 2.09 ab	15.11 ± 0.94 c	26.30 ± 0.97 ab	57.56 ± 7.29 bc	4.03 ± 0.19 ab	13.99 ± 1.29 a
AP 0.05%	56.99 ± 2.91 b	14.41 ± 0.6 bc	37.27 ± 16.28 b	63.01 ± 0.81 c	4.03 ± 0.19 ab	14.94 ± 0.47 a
AP 0.1%	55.44 ± 1.46 ab	15.58 ± 0.77 c	27.17 ± 0.24 ab	58.4 ± 0.41 bc	3.65 ± 0.51 a	13.54 ± 0.21 a
WControl	56.30 ± 1.07 b	12.36 ± 0.24 a	22.14 ± 0.39 a	51.25 ± 3.14 ab	8.77 ± 0.17 c	17.22 ± 0.81 b
WAA 0.01%	56.91 ± 0.04 ab	13.05 ± 0.72 ab	23.31 ± 1.19 a	51.18 ± 0.67 ab	8.79 ± 0.24 c	17.3 ± 0.05 b
WAP 0.05%	57.99 ± 1.42 b	13.32 ± 0.35 ab	24.82 ± 0.55 ab	48.14 ± 5.29 a	8.56 ± 0.57 c	16.89 ± 1.16 b
WAP 0.1%	55.97 ± 1.43 ab	13.53 ± 0.03 ab	24.22 ± 0.5 ab	48.66 ± 2.57 a	8.53 ± 0.39 c	17.06 ± 0.73 b

Control: wheat flour; AA 0.01%: wheat flour with 0.01% ascorbic acid; AP 0.05%: wheat flour with 0.05% acerola powder; AP 0.1%: wheat flour with 0.1% acerola powder; WControl: whole wheat flour; WAA 0.01%: whole wheat flour with 0.01% ascorbic acid; WAP 0.05%: whole wheat flour with 0.05% acerola powder; WAP 0.1%: whole wheat flour with 0.1% acerola powder. Values with the same letter in the same parameter do not present significant differences (*p* < 0.05).

## Data Availability

Not applicable.
